# Dengue and Chikungunya Co-Infection-Associated Multi-Organ Dysfunction Syndrome: A Case Report

**DOI:** 10.7759/cureus.50196

**Published:** 2023-12-08

**Authors:** Vikram B Vikhe, Ahsan A Faruqi, Avani Reddy, Devansh Khandol, Vivek H Lapsiwala

**Affiliations:** 1 General Medicine, Dr. D. Y. Patil College, Hospital and Research Centre, Dr. D. Y. Patil Vidyapeeth, Pune, IND

**Keywords:** thrombocytopenia, dengue fever (df), dengue hemorrhagic fever (dhf), diffuse alveolar hemorrhage, hepatitis, co-infection, multi-organ dysfunction, encephalitis, chikungunya, dengue

## Abstract

Dengue and chikungunya infections are increasing globally, especially in India. While the majority of patients settle with symptomatic management, some develop life-threatening complications. Here we discuss a case of co-infection between dengue virus (DENV-2) and chikungunya virus (CHIKV) in a young Indian male who presented with an acute febrile illness that progressed to multi-organ dysfunction involving the hepatic, nervous, respiratory, and hematological systems. We discuss the management of this complicated case and attempt to generate awareness regarding the severity of co-infection by these viruses.

## Introduction

Dengue and chikungunya cases and related deaths have been rising year by year worldwide, especially in India [1-3]. Aedes mosquitoes are the common vector for the spread of both infections. While dengue fever is caused by an RNA virus known as dengue virus (DENV) with four distinct but closely related antigenic serotypes (DENV-1 to DENV-4), chikungunya fever, on the other hand, is caused by an RNA virus known as chikungunya virus (CHIKV) and has no such serotypes [1].

The clinical spectrum of dengue varies from low-grade fever and rash to serious side effects such as dengue hemorrhagic fever (DHF) and shock. Severe thrombocytopenia and plasma leakage leading to hemoptysis, nasal bleeding, gum bleeding, hematuria, and melena are common signs of DHF [4]. Respiratory distress and hemoptysis with a chest x-ray showing new infiltrates in a dengue patient should raise suspicion of diffuse alveolar hemorrhage (DAH), which can be a dreaded complication of DHF [5]. Dengue-associated multi-organ dysfunction syndrome has been described as the involvement of two or more organs or systems manifested as a combination of complications, such as hepatitis, DAH, encephalitis, myocarditis, disseminated intravascular coagulation, and shock [6].

Chikungunya can present as an acute, subacute, or chronic illness. Clinically, the illness is marked by an acute febrile illness, sometimes with severe and debilitating arthralgia or arthritis that lasts for a variable amount of time. Reports have also shown neurological consequences, including meningoencephalitis. Usually, patients recover fully with long-lasting immunity [7].

DENV and CHIKV co-infection have been associated with a more severe clinical disease than with either of them alone, with an overall higher need for mechanical ventilation and a higher mortality rate. Although dengue is more likely to cause serious consequences, including death, chikungunya is often not lethal. Hence, coinfection may cause sickness with overlapping signs and symptoms, which complicates the physician's diagnosis and course of treatment [8].

## Case presentation

A 22-year-old Indian male presented to our outpatient clinic with an acute febrile illness for three days accompanied by retro-orbital discomfort, jaundice, and generalized body pain. On admission, he was febrile with a body temperature of 101.2 °F, pulse rate of 94 beats/min, blood pressure of 120/70 mmHg, respiratory rate of 16 cpm, and oxygen saturation of 98% on room air. Upon examination, there was a reduction in skin turgor, indicating dehydration, and mild tenderness was noted in the right upper quadrant with hepatomegaly. The patient was investigated for the common causes of acute febrile illness that are associated with hepatic impairment, like dengue, chikungunya, malaria, and rickettsia. Reports on day 1 (Tables 1, 2) were suggestive of dengue and chikungunya co-infection fever with hepatitis and thrombocytopenia. Fluid resuscitation with crystalloids for dehydration and an infusion of n-acetylcysteine (NAC) (100 mg/kg/day) for hepatitis were initiated [9].

**Table 1:** Blood workup from day 1 to day 11 of the patient D: Day; SGOT: serum glutamic-oxaloacetic transaminase; SGPT: serum glutamic pyruvic transaminase

Parameters (normal limit)	Day1	D3	D4	D5	D7	D9	D10	D11
Hemoglobin (13.2-16.6 gm/dl)	13	12.2	9.2	10.2	11.2	12.00	12.8	12.80
Total leucocyte count (4,000-10,000 /µL)	3300	2200	2200	2800	3400	3800	4200	4600
Platelets (1,50,000-4,10,000 /µL)	46,000	13,000	9,000	13,000	55,000	99,000	1.45L	2.24L
Serum urea (17–49 mg/dL)	28	30	53	55	40	33	30	30
Serum creatinine (0.6–1.35 mg/dL)	0.77	0.90	0.90	0.92	0.90	0.80	0.78	0.78
Serum bilirubin (0.2–1.2 mg/dL)	8.03	5.02	5.92	4.80	3.80	2.80	1.2	1.1
SGOT (8–48 IU/L)	1454	654	443	223	133	99	47	47
SGPT (7–55 IU/L)	1654	554	394	221	123	88	53	52
Random blood sugar level (up to 140mg/dl)	166	116	123	183	163	140	110	108

**Table 2:** Additional laboratory reports DENV: Dengue virus; CHIKV: Chikungunya virus

Test	Result
Anti-Dengue IgM	Positive
Anti-Dengue IgG	Negative
Dengue RT-PCR	Positive(DENV-2)
Anti-CHIKV IgM	Positive
Anti-CHIKV IgG	Negative
CHIKV RT-PCR	Positive
Rapid malaria test	Negative
Weil Felix test	Negative
HIV	Negative
Hepatis A,B,C,E	Negative

**Figure 1:**
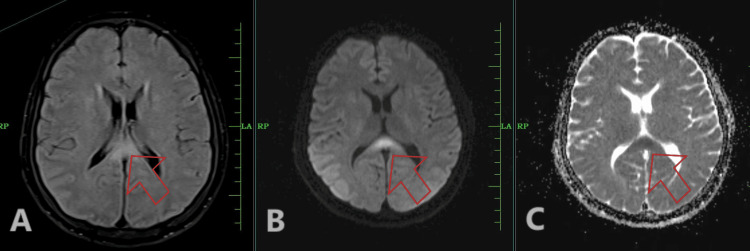
MRI brain of the patient showing encephalitis changes An area of altered signal intensity noted in the splenium of the corpus callosum (shown by red arrow), appearing hyperintense on T2-weighted image sequence (A), showing diffusion restriction on diffusion-weighted imaging sequence (B) with corresponding low apparent diffusion coefficient value (C).

**Figure 2:**
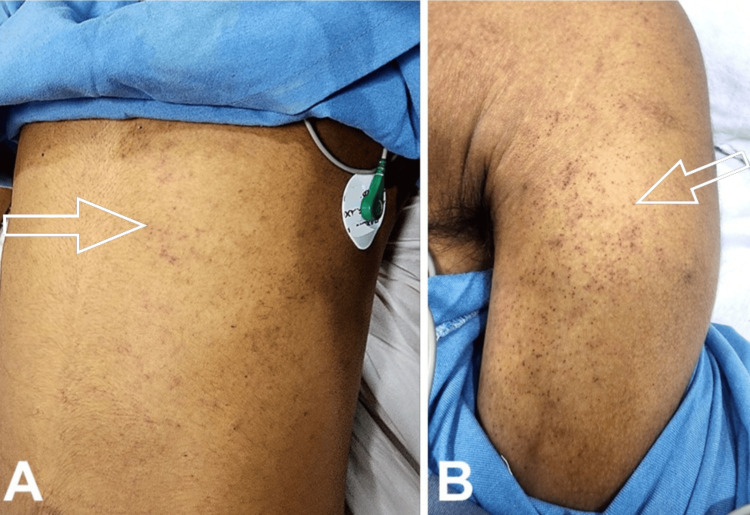
Petechiae can be seen on the patient's trunk (A) and left arm (B) shown by white arrow

**Figure 3:**
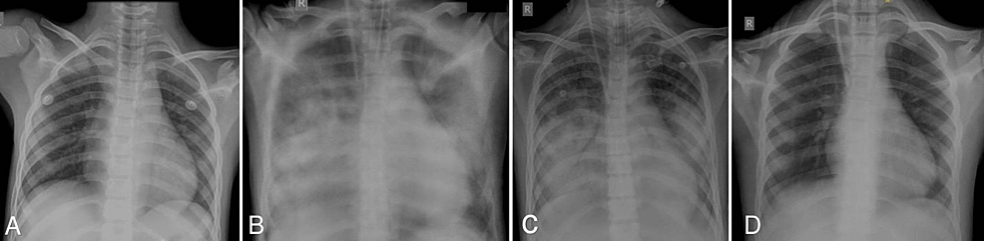
Chest x-ray radiographs from day 1 to day 10 (A) Day 1: No obvious abnormality; (B) Day 4: Multiple new bilateral opacities; (C) Day 6: Bilateral opacities resolving; (D) Day 10: Resolution of opacities

**Figure 4:**
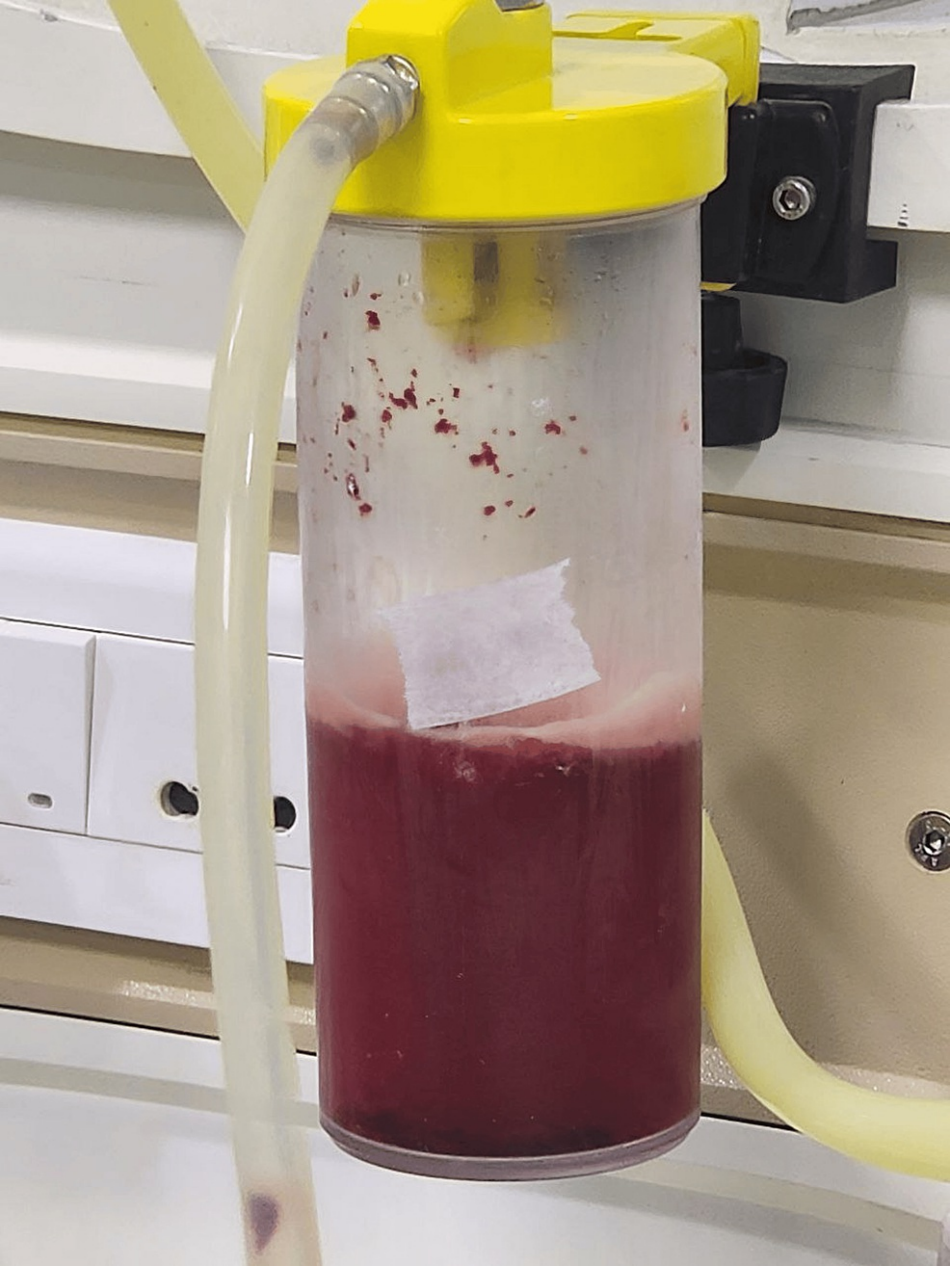
Suction collection tube of the patient containing blood tinged secretions

On the early morning of day 3, the patient had an episode of generalized tonic-clonic seizure associated with involuntary micturition, which was terminated by a stat dose of intravenous (iv) lorazepam. Postictal confusion was present. MRI brain (Figures 1A-1C) and lumbar puncture of the patient showed viral encephalitis changes. A loading dose of injection levetiracetam of 1 g IV stat, followed by 500 mg IV BD, along with an injection of Acyclovir 10 mg/kg IV TDS, was initiated. As the thrombocytopenia of the patient worsened [Table 1], petechiae developed over the trunk and all limbs (Figures 2A, 2B). The same evening, the patient had an episode of hemoptysis of approximately 10 mL along with a fall in saturation, for which oxygen support was commenced, and single donor platelets along with fresh frozen plasma and packed red blood cells were transfused. Multiple new bilateral ground-glass opacities on the patient's chest x-ray (Figures 3A-3D) raised suspicion of DAH. Immediate endotracheal intubation was performed, and mechanical ventilator support was commenced. Injection cefepime 1 g IV BD was added for prophylaxis of ventilator-associated infections. Blood-tinged secretions were noted in the endotracheal and oral suctions (Figure 4). The patient was sedated, paralyzed, and started on a high positive end-expiratory pressure (PEEP) with a low tidal volume (TV) approach for DAH. Inj. Methylprednisolone 1 g IV OD was administered for three days, followed by 1 mg/kg/day, and then tapered later via tablet prednisolone. The patient was kept sedated on ventilator support, with arterial blood gases and other laboratory parameters being monitored for the next five days (Tables 1, 3).

**Table 3:** Arterial blood gas analysis of the patient ABG: arterial blood gas; pCO_2_: partial pressure of carbon dioxide; pO_2_: partial pressure of oxygen; SpO_2_: arterial oxygen saturation; sHCO_3-_: serum bicarbonate; FiO_2_: Fraction of inspired oxygen

ABG	D4	D5	D6	D7	D8	D9	D10
pH (7.350–7.450)	7.23	7.34	7.48	7.42	7.45	7.40	7.40
pO_2_ (83.0–108 mmHg)	53	90	115	130	118	90	85
pCO_2_ (35.0–45.0 mmHg)	40	40	50	48	46	40	40
sHCO3 - (18–24 mmol/L)	18	24	29	26	24	23	23
SpO_2_ Saturation	90%	92%	100%	100%	100%	100%	99%
FiO_2_	0.60	1	1	0.80	0.60	0.30	Room air (0.21)
PO2/FiO2	88	90	115	162	196	300	404

**Figure 5:**
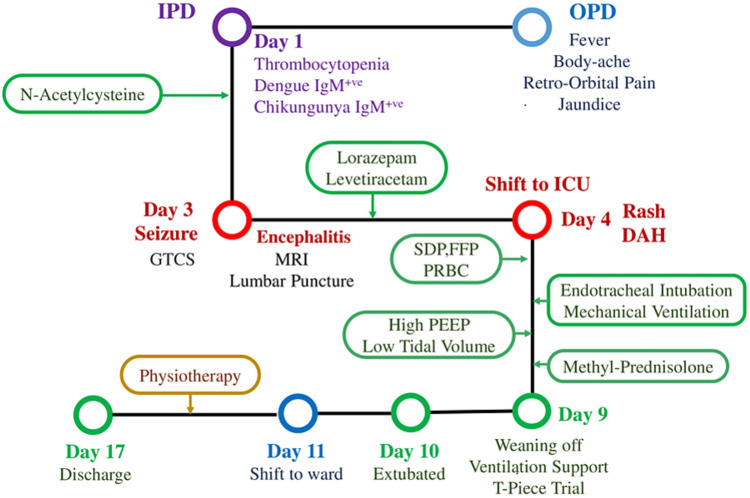
Timeline of events from OPD visit to discharge OPD: Out-Patient Department; IPD: In-Patient Department; GTCS: Generalized Tonic Clonic Seizure; ICU: Intensive Care Unit; DAH: Diffuse Alveolar Hemorrhage; S.D.P: Single Donor Platelets; F.F.P: Fresh Frozen Plasma; PRBC: Packed Red Blood Cells; PEEP: Positive End Expiratory Pressure

The patient was weaned from the mechanical ventilator, and a T-piece trial was given, followed by extubation. The patient was then shifted to the ward for observation and was discharged after a week of physiotherapy (Figure 5). The follow up has been uneventful.

## Discussion

Coinfection of DENV and CHIKV is common in India, and most coinfections are associated with a poorer outcome than mono-infection [8,10]. Both infections affect the liver, and the development of acute liver dysfunction in the early course of the disease has been implicated as a warning sign of severe disease. The pathogenesis of liver injury in these infections is not clearly understood. Some theories include a direct viral or host-generated immune response causing injury to liver cells and localized vascular leaks inside the liver [11]. In addition, antibody-dependent enhancement shown by both viruses results in the augmentation of viral replication by sub-neutralizing antibodies, thereby exacerbating disease severity [12]. NAC exhibits potential for treating dengue-induced acute liver disease through multiple mechanisms. Its antioxidant properties, as a precursor to glutathione, may reduce oxidative stress, while its anti-inflammatory effects could alleviate liver inflammation caused by the virus. Additionally, NAC's known hepatoprotective qualities and potential to enhance liver blood flow might aid in protecting liver cells from damage and improving their recovery [9].

The DENV serotypes DENV-2, DENV-3, and CHIKV, which are neurotropic, although rare, are associated with several neurological complications such as encephalitis, Guillain-Barré syndrome, transverse myelitis, optic neuritis, and acute disseminated encephalomyelitis. The invasion of DENV and CHIKV triggers an immune response that can misfire, activating autoimmune factors that inadvertently attack neural tissues. Simultaneously, these infections induce metabolic changes that disrupt normal neural function, collectively contributing to the neurological complications associated with these viruses [13].

Severe dengue virus infection symptoms are linked to an aggressive inflammatory response known as a cytokine storm, which is the concurrent production of large amounts of proinflammatory cytokines accompanied by abnormalities in the coagulation cascade, platelet dysfunction, impaired membrane permeability, and direct injury to the alveolar lining cells by DENV, ultimately causing the development of DAH [14]. The management of DAH in intubated patients includes the use of high PEEP and low TV (6 mL/kg/ideal body weight) for combating refractory hypoxemia, along with the administration of pulse methylprednisolone (1g IV OD) unless a non-immune cause of DAH is apparent [15].

## Conclusions

In regions endemic to dengue and CHIKVs, while single infections are more prevalent, the emergence of coinfections necessitates physicians to routinely test for both viruses in cases of acute febrile illness. Maintaining a watchful approach, especially with co-infections, is crucial due to their potential to escalate to severe complications like DAH, hepatitis, and encephalitis, posing life-threatening risks if not promptly addressed. Since no specific treatments exist for these viruses, management primarily relies on attentive, supportive care and close monitoring of the patient's condition. Early identification and intervention are pivotal in preventing adverse outcomes in such cases.
